# Antibacterial Efficacy of Calcium Hypochlorite with Vibringe Sonic Irrigation System on* Enterococcus faecalis*: An In Vitro Study

**DOI:** 10.1155/2016/8076131

**Published:** 2016-04-27

**Authors:** Aysin Dumani, Hatice Korkmaz Guvenmez, Sehnaz Yilmaz, Oguz Yoldas, Zeliha Gonca Bek Kurklu

**Affiliations:** ^1^Department of Endodontics, Faculty of Dentistry, Cukurova University, 01330 Adana, Turkey; ^2^Art and Science Faculty, Department of Biology, Cukurova University, 01330 Adana, Turkey

## Abstract

*Aim*. The purpose of this study was to compare the in vitro efficacy of calcium hypochlorite (Ca[OCl]_2_) and sodium hypochlorite (NaOCl) associated with sonic (Vibringe) irrigation system in root canals which were contaminated with* Enterococcus faecalis*.* Material and Methods*. The root canals of 84 single-rooted premolars were enlarged up to a file 40, autoclaved, inoculated with* Enterococcus faecalis*, and incubated for 21 days. The samples were divided into 7 groups according to the irrigation protocol: G0: no treatment; G1: distilled water; G2: 2.5% NaOCl; G3: 2.5% Ca(OCl)_2_; G4: distilled water with sonic activation; G5: 2.5% NaOCl with sonic activation; and G6: 2.5% Ca(OCl)_2_ with sonic activation. Before and after decontamination procedures microbiological samples were collected and the colony-forming units were counted and the percentages of reduction were calculated.* Results*. Distilled water with syringe irrigation and sonic activation groups demonstrated poor antibacterial effect on* Enterococcus faecalis* compared to other experimental groups (*p* < 0.05). There was no statistically significant difference between syringe and sonic irrigation systems with Ca(OCl)_2_ and NaOCl.* Conclusion*. The antimicrobial property of Ca(OCl)_2_ has been investigated and compared with that of NaOCl. Both conventional syringe irrigation and sonic irrigation were found effective at removing* E. faecalis* from the root canal of extracted human teeth.

## 1. Introduction 

The major aim of the root canal treatment is to effectively clean and disinfect the root canal system to eliminate microorganisms [1]. However, during root canal therapy the complex anatomy of the root canal system prevents the penetration of irrigants and medicaments, so resistant bacteria can remain within the canal system and reinfect the root canal if they are not eliminated [2].* Enterococcus faecalis* is one of these microorganisms and has the ability to penetrate as far as 250 *μ*m into the dentinal tubules, which provides showing resistance to irrigation solutions usually used during the instrumentation of root canals [3].

There are limitations of conventional syringe irrigation to reach the ramifications and morphologic irregularities of the root canal system [4]. To improve root canal disinfection, researchers have introduced alternative procedures like ultrasonic, sonic, brush-covered needles, manual dynamic activation, and aspiration/irrigation systems [5, 6]. Vibringe sonic irrigation system supplies delivery and activation of the irrigation solution in the root canal, in only one step. The activation of the disinfectant by acoustic streaming may improve the disruption of the biofilm and be useful for cleaning complex anatomic areas [7].

Several endodontic irrigants have been used in root canal therapy but sodium hypochlorite (NaOCl) is the most commonly used endodontic irrigant because of its excellent antibacterial property and its ability to dissolve organic tissue [4]. However, there is still controversy regarding which concentration of the solution would be most efficacious against the microorganisms and still safe for the patient [8]. There is resurgence of interest in finding alternative endodontic irrigant with the same efficacy as NaOCl, but this new irrigant should preferably have less toxicity.

Calcium hypochlorite (Ca(OCl)_2_) shows antibacterial properties [9] and the ability to promote soft-tissue dissolution in the same level of NaOCl [10]. This solution is normally used for industrial sterilization and water purification treatments [11] and there is a unique study in the literature evaluating the antimicrobial potential of this solution against* E. faecalis* in root canals [12]. To our knowledge, there is no study in the literature related to antibacterial effect of Vibringe sonic irrigation system with Ca(OCl)_2_. Therefore, the aim of this study was to compare the efficacy of sonic and syringe irrigation of NaOCl and Ca(OCl)_2_ on* Enterococcus faecalis* in extracted human teeth.

## 2. Material and Methods

### 2.1. Selection and Preparation of Teeth

This study was approved by the University of Cukurova Institutional Review Board. Eighty-four extracted, single-rooted premolars were obtained from patients affected by dental caries or severe periodontal disease. Bone and calculus on the root surface were removed with curettes. The teeth were standardized to a length of 14 mm with a diamond bur. The working length (WL) was set at 13 mm, 1 mm short of the anatomical apex. The patency of each canal was established with a size 15 K-file. Size 4 and 3 Gates Glidden Burs (Dentsply Maillefer) were used to flare the coronal aspect of each canal and the root canal preparations were performed with a WaveOne (Dentsply Tulsa Dental Specialties, OK, USA) rotary system #40 file size at the WL according to the manufacturers' instructions. During instrumentation, irrigation was performed with 5 mL of 2.5% NaOCl using side-vented needles (0.3 × 25 Endo-Top, PPH Cerkamed, Poland). After instrumentation, the root canals were irrigated with 3 mL of 17% EDTA (Calasept, Nordiska Dental, Sweden) and then filled with 1 mL of 17% EDTA for three minutes to remove the smear layer. Final irrigation was performed with 2 mL of distilled water (DW). The root canals were dried with paper points, composite resin (3M, Saint Paul, MN, USA) was used to seal the apex, and nail polish was applied in two layers around the root surface to prevent bacterial leakage. Each root was fixed with Putty-C Silicone for impression (Panasil Putty Soft, Kettenbach LP, USA) in an Eppendorf tube to facilitate handling and identification. The tubes were placed in plastic carrier boxes, placed in autoclave sachets, and then autoclaved for 30 minutes at 121°C (Hirayama, HV-50L, Japan). Sterilization was checked with an indicator (4A Medical, Emulating Indicator, Ankara, Turkey) placed in the sachets. One investigator was assigned to perform all of the procedures (Aysin Dumani).

### 2.2. Contamination of the Specimens

*E. faecalis* (ATCC 29212) were obtained from the culture collection at Refik Saydam Hıfzısıhha Institute, Ankara, Turkey. The bacteria had previously been cultured on 5% blood agar (supplemented with 5% sheep blood, bioMerieux, Turkey) plates and cultured in brain-heart infusion broth (BHI, bioMerieux, Turkey). Bacterial strains were confirmed by Gram stain and by colonial and growth characteristics.* E. faecalis* colonies were harvested from the blood agar and suspended in 4 mL of PBS (Phosphate-Saline Buffer). The microorganism suspension was diluted with sterile PBS to achieve 10^8^ CFU/mL using McFarland standard tubes. Then 84 sterilized root canals were opened under a laminar flow hood (Herasafe, Thermo Scientific, Germany) and filled with 20 *μ*L of* E. faecalis* suspension using sterile automatic micropipettes (Pipetman Neo, Gilson, France). Sterile size 15 K-files were used to carry the bacterial suspension to the WL. All specimens were incubated at 37°C for 21 days, and the root canal contents were replaced with fresh and sterile BHI every 48 hours. At seven-day intervals, sampling was performed from five randomly selected teeth and tested for bacterial contamination. The bacterial sample was submitted to Gram staining and cultured on blood agar (for hydrolysis control) and Na-Azide agar (selective medium for* E. faecalis*, bioMerieux), followed by catalase and esculin tests.

After the incubation period, initial samples were collected with three sterile paper points of size 35. Each paper point remained in the canal for one minute. The absorbent paper points were transferred to a tube containing 2 mL sterile PBS and vortexed for 30 seconds. The sample was diluted to 10^−4^ in PBS and 0.1 mL of the 10^−4^ dilutions was plated on blood agar. The plates were then incubated at 37°C for 24–48 hours. The bacterial count was measured in CFU/mL and was performed using an IUL colony counter (IUL, S.A., Barcelona, Spain).

### 2.3. Preparation of Irrigation Solutions

The Ca(OCl)_2_ solution was made up from granules (Jianghan Salt & Chemical Complex, Qianjiang, Hubei, China) at the time of the experiment. The desired concentration of 2.5% Ca(OCl)_2_ solution was prepared using distilled water (weight/volume (w/v) ratio) and mixed with a magnetic stirrer for 30 minutes. The NaOCl solution (chloraxid, PPH Cerkamed, Poland) was prepared at 2.5% concentration using distilled water.

### 2.4. Classification of Treatment Group

After the period of incubation, 12 contaminated specimens served as a control group for the inoculation and incubation process and remained untreated. The other 72 specimens were divided into the following six treatment groups (G) (*n* = 12 per group):

G1: syringe irrigation (SI) with sterile distilled water (DW) (SI + DW).
G2: SI with 2.5% NaOCl solution (SI + NaOCl).
G3: SI with 2.5% Ca(OCl)_2_ solution (SI + Ca(OCl)_2_).
G4: Vibringe sonic irrigation system (VIS) with DW (VIS + DW).
G5: VIS with 2.5% NaOCl (VIS + NaOCl).
G6: VIS with 2.5% Ca(OCl)_2_ (VIS + Ca(OCl)_2_).

For syringe irrigation groups, a side-vented needle tip was placed at the 12 mm, and 5 mL of irrigant was delivered over a period of approximately 2 minutes. For sonic irrigation groups, 5 mL irrigation solution was delivered and sonically activated for 2 minutes at −1 mm from the WL with the Vibringe system by using a Luer-Lock syringe (B.V. Corp., Amsterdam, Netherlands) and side-vented needle according to the manufacturer's instructions. For each group all procedures were performed under aseptic conditions in a laminar flow hood with sterile gloves and sterile needles for each sample. After irrigation protocols, groups with NaOCl and Ca(OCl)_2_ irrigation solution were irrigated with 3 mL 5% sodium thiosulfate for one minute to inactivate the residual effect of these irrigants and the other groups were irrigated with 3 mL sterile DW for one minute. One investigator was assigned to perform all of the irrigation protocols (Aysin Dumani).

### 2.5. Microbiologic Analysis

After irrigation of the root canals, a number 35 H-file with the handle cut-off was introduced to the WL with a discrete filing motion and this H-file was transferred to a tube containing 1 mL of sterile PBS and vortexed for one minute. If the root canal was dry, sterile distilled water was introduced into the canal. Three sterile paper points (size 35) were placed in the canal for one minute each and transferred to the same tubes of the file. After being vortexed for one minute the same culturing procedures were applied as for the initial sampling. The bacterial count in CFU/mL was calculated.

### 2.6. Statistics

The log transformation of each CFU/mL count was performed, and statistical tests were applied. The paired *t*-test was used for intragroup analysis, and intergroup analysis was performed using analysis of variance test. The level of significance for all analyses was set at *p* < 0.05.

## 3. Results

The results from the control group showed colony counts of about 10^5^ CFU/mL, demonstrating a decrease of three log steps through the inoculation and incubation processes and the further processing of the samples. The logarithmic mean and SD of bacterial counts (CFUs/mL) of each group before and after irrigation protocols and bacterial reductions in different groups are presented in Table 1.

There was no significant difference between the initial bacterial counts in all groups. The bacterial load was homogeneous before the irrigation protocols were performed (*p* > 0.05). Distilled water with syringe irrigation and sonic activation groups demonstrated poor antibacterial effect on* Enterococcus faecalis* compared to other experimental groups (*p* < 0.05). There was no statistically significant difference between syringe and sonic irrigation systems with Ca(OCl)_2_ and NaOCl (*p* > 0.05).

## 4. Discussion 

The model of bacterial growth used in this study has already been reported in previous studies that investigated antimicrobial strategies against* E. faecalis* [12, 13]. This microorganism was chosen because of its high resistance to conventional endodontic procedures and low nutrient requirements [14]. The specimens were infected with* E. faecalis* for 21 days in this study, because this microorganism invades dentinal tubules after 21 days of incubation [15].

In the literature, there is no consensus on evaluating the bacterial reduction. Some studies calculated bacterial reduction as the difference between before and after treatment by counting the CFUs [12]. In contrast, other studies reported the inoculated bacteria as microliter with no data on bacteria count [16, 17]. Thus, methodological differences may represent variable results in bacterial reduction values. In the present study, initial bacterial samples were taken from root canals with paper points and incubated in blood agar plates. The colonies grown on the blood agar were counted and interpreted as CFUs/mL.

In order to evaluate the bacterial count, CFUs were expressed in log CFU/mL in the present study. This method was chosen based on previous studies and it allows, in an acceptable way, bacterial quantification from the root canal [12, 16]. The plate culture method is the most commonly used methodology in bacterial reduction studies. However, sampling procedure is very determinative that could influence the results. The scraping sampling technique may help to collect the bacteria from the smear layer, biofilm remnants, and noninstrumented areas [17]. Thus, in this study after irrigation protocols samples were collected from the root canals with H-files and paper points.

Several irrigation solutions have been used in endodontic treatment to promote an adequate decontamination of the root canal system. Although NaOCl is the most preferred irrigant by clinicians, there is a considerable finding in the literature regarding the antibacterial effect of NaOCl for* E. faecalis* [18, 19]. NaOCl was not regarded as optimum for this microorganism because of its penetration ability of dentinal tubules and resistance to intracanal medication. On the other hand, because of the adverse effects such as influencing negatively the bond strength between adhesive restorations and dentin and reducing the resistance of teeth to fracture, researchers have focused on new endodontic irrigants [20]. Ca(OCl_2_) is a chemical substance that is commonly used for industrial sterilization and water purification treatments [11]. However, limited studies were conducted with Ca(OCl_2_) as an endodontic irrigant [12, 21]. There is only one study about the antibacterial activity of Ca(OCl_2_) and it has shown that Ca(OCl_2_) eliminated* E. faecalis* from the root canals as well as NaOCl with or without passive ultrasonic irrigation [12]. The influence of passive ultrasonic irrigation to deliver Ca(OCl_2_) and the antimicrobial activity against* E. faecalis* have been investigated in the mentioned study. The aim of this study was to investigate the effectiveness of Vibringe, sonically activated delivery system, and syringe irrigation of NaOCl and Ca(OCl)_2_ on* E. faecalis* in extracted human teeth.

Calcium hypochlorite was expected to have more antimicrobial efficacy than sodium hypochlorite because of the higher generation of hypochlorous acid when mixed with water [21]. Hypochlorous acid is responsible for the antibacterial activity by disruption of several vital functions of the microbial cell [22]. Consistent with this result, in this study the antimicrobial property of 2.5% Ca(OCl)_2_ was effective as 2.5% NaOCl with two irrigation systems and showed 99.9% reduction of intracanal 21-day-old* E. faecalis*. However, 2.5% concentrations of Ca(OCl)_2_ and NaOCl were not adequate for complete eradication of* E. faecalis* in the samples. Increasing the concentration of irrigant may improve the antibacterial action and the percentage of bacterial reduction, but the risk of cytotoxicity can increase in higher concentrations [23]. The cytotoxic potential of NaOCl had been known but there is currently no research about Ca(OCl)_2_ cytotoxicity and how it may affect the periapical tissues in in vivo conditions.

In addition to comparing the antibacterial effectiveness of these irrigants, different irrigation systems were compared to eliminate* E. faecalis* in this study. Syringe irrigation with needles is the standard procedure but, unfortunately, it is not effective in the apical root canal or in isthmuses or oval extensions [24]. Therefore the use of sonic or ultrasonic agitation of irrigants is expected to improve irrigation dynamics and bacterial elimination from root canal systems. Conflicting results have been published regarding the effectiveness of sonic activation of the irrigant to remove bacteria [12, 25–27]. Bago et al. [25] and Seet et al. [26] demonstrated that sonic irrigation (Endoactivator) of NaOCl was more successful in reducing root canal infection than NaOCl with syringe irrigation. However, Tardivo et al. [27] and de Almeida et al. [12] found no statistically significant difference between ultrasonic activation and conventional syringe irrigation to eliminate* E. faecalis*. Consistent with these studies, the current research showed that sonic irrigation system did not improve the potential for decontamination in root canals infected with* E. faecalis* and findings were not statistically different from syringe irrigation.

This may be attributed to removal of debris and smear layer which are likely to harbor bacteria [28]. Although it is known that Ca(OCl)_2_ was ineffective in removing the smear layer [21], Vibringe sonic irrigation system also showed inconsistent results when evaluating smear and debris removal [7, 29, 30]. Rödig et al. [7] and Kumar et al. [29] demonstrated that sonic irrigation system removed debris significantly better than syringe irrigation in only apical root canal third, whereas Johnson et al. [30] concluded that there is no difference between the Vibringe and side-vented needle irrigation in their overall debridement efficacy in apical one-third of the mesiobuccal root of maxillary first molars.

Complete eradication of* E. faecalis* was observed in few of the samples in this study. This microorganism has the ability to maintain viability in obturated root canals [14]; therefore more effort needs to be done to achieve the goal of complete elimination of infection from the root canal. In further studies, this sonic irrigation system could be used during instrumentation of the root canals with different amount and concentration of irrigation solutions. Besides the antimicrobial effectiveness of different concentration of Ca(OCl)_2_ with longer irrigation times, cytotoxicity and the interactions with other root canal irrigants should be investigated in future studies.

## 5. Conclusion

The antimicrobial property of 2.5% Ca(OCl)_2_ was as effective as 2.5% NaOCl. Sonically activated irrigation with Vibringe system did not improve the antibacterial effectiveness of NaOCl and Ca(OCl)_2_.

## Figures and Tables

**Table 1:** Bacterial reduction and counts of *E. faecalis* (in log) before and after irrigation protocols.

Group	Before	After	Bacterial reduction		*p* ^†^	*p* ^‡^
	Mean ± SD	Mean ± SD	Mean ± SD	%		
G1 (SI + DW)	5.40 ± 0.16	4.90 ± 0.40	0.50 ± 0.36	59.88	0.001	a
G2 (SI + NaOCl)	5.52 ± 0.18	1.71 ± 0.87	3.80 ± 0.83	99.96	<0.001	b
G3 (SI + Ca(OCl)_2_)	5.35 ± 0.31	1.69 ± 0.87	3.65 ± 0.88	99.93	<0.001	b
G4 (VIS + DW)	5.43 ± 0.27	4.96 ± 0.48	0.47 ± 0.30	59.42	<0.001	a
G5 (VIS + NaOCl)	5.50 ± 0.22	1.62 ± 0.98	3.88 ± 1.14	99.96	<0.001	b
G6 (VIS + Ca(OCl)_2_)	5.54 ± 0.22	1.64 ± 1.04	3.90 ± 0.96	99.96	<0.001	b

^†^Significant difference according to the paired *t*-test (*p* < 0.05).

^‡^The means for groups in heterogeneous subsets are displayed with the different letters and illustrate the significant difference according to analysis of variance for treatment groups (*p* < 0.05).
